# Supplementary material for: Morinda citrifolia Essential Oil: A Plant Resistance Biostimulant and a Sustainable Alternative for Controlling Phytopathogens and Insect Pests
Source: Biology (Basel). 2024 Jun 27;13(7):479. doi: 10.3390/biology13070479 (PMC11274064; doi:10.3390/biology13070479)
Supplement: Supplementary file 1 [file biology-13-00479-s001.zip › biology-3041344-supplementary.pdf]

Figure S1: Chromatographic profile (CG-MS) from *Morinda citrifolia* essential oil.

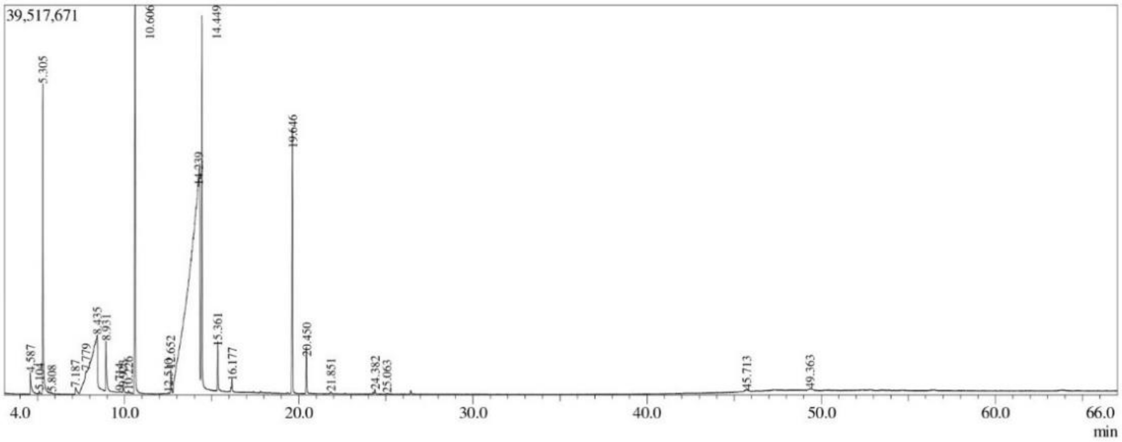

Peak Report

Peak#	R.Time	I.Time	F.Time	Area	Area%	Height	Height%	A/H	Mark	Name
1	4.587	4.550	4.850	9397104	0.57	2168663	1.21	4.33	S	2,3-Pentanedione, 4-methyl-
2	5.104	5.070	5.135	501335	0.03	275635	0.15	1.82		Cyclopentane, 1,2,3,4,5-pentamethyl-
3	5.305	5.265	5.855	76180862	4.63	31401295	17.53	2.43	S	Hexanoic acid, methyl ester
4	5.808	5.680	5.850	288324	0.02	87677	0.05	3.29	T	Sulfurous acid, cyclohexylmethyl dodecyl
5	7.187	7.145	7.395	4414771	0.27	670765	0.37	6.58		Hexanoic acid, ethyl ester
6	7.779	7.395	7.810	25807142	1.57	2265747	1.26	11.39	V	Butanoic acid, 4-pentenyl ester
7	8.435	7.810	8.800	155697720	9.46	5850535	3.27	26.61	V	Hexanoic acid
8	8.931	8.800	9.360	21861527	1.33	5170751	2.89	4.23	V	Butanoic acid, 4-pentenyl ester
9	9.714	9.630	9.870	676421	0.04	136167	0.08	4.97		2-Nonanone
10	9.923	9.870	10.110	2238445	0.14	534405	0.30	4.19		(E)-2-Methylbut-2-en-1-yl isobutyrate
11	10.226	10.160	10.300	658390	0.04	226312	0.13	2.91		Butanoic acid, 3-methyl-, 3-methyl-3-buter
12	10.606	10.520	10.940	119517005	7.26	38747046	21.63	3.08		Octanoic acid, methyl ester
13	12.510	12.440	12.600	310257	0.02	65173	0.04	4.76		Hexanoic acid, hexyl ester
14	12.652	12.600	12.780	7474099	0.45	2407412	1.34	3.10	V	Octanoic acid, ethyl ester
15	14.239	12.780	14.355	962100285	58.43	21391914	11.94	44.97	V	Octanoic acid
16	14.449	14.355	15.155	134516272	8.17	35214681	19.65	3.82	V	Hexanoic acid, 4-pentenyl ester
17	15.361	15.155	16.080	21612500	1.31	4486999	2.50	4.82	SV	Hexanoic acid, 3-methyl-2-butenyl ester
18	16.177	16.080	16.505	5589602	0.34	1217982	0.68	4.59	V	Decanoic acid, methyl ester
19	19.646	19.505	20.030	75803225	4.60	21757413	12.14	3.48		Isobutyl 3-methylbut-3-enyl carbonate
20	20.450	20.355	20.630	13296233	0.81	4071126	2.27	3.27		Octanoic acid, 3-methylbut-2-enyl ester
21	21.851	21.705	22.055	887676	0.05	192548	0.11	4.61		Hexanoic acid, phenylmethyl ester
22	24.382	24.205	24.580	2163605	0.13	452111	0.25	4.79		Dodecanoic acid, 4-penten-1-yl ester
23	25.063	25.005	25.280	490758	0.03	55694	0.03	8.81		Cyclopentanecarboxylic acid, 3-tridecyl es
24	45.713	45.455	45.955	2726555	0.17	176654	0.10	15.43		9,19-Cycloergost-24(28)-en-3-ol, 4,14-dim
25	49.363	49.155	49.655	2402490	0.15	151805	0.08	15.83		25-Nor-9,19-cyclolanostan-24-one, 3-acet
				1646612603	100.00	179176510	100.00			

Table S1. Summary of the linear and/or non-linear regression analyses (enzymes) of the curves shown in figure 2

enzyme	Modelo	Treatment	Estimated parameters (±SD)			df_{error}	*F*	*P*	R^2
			**a**	**b**	**x0/y0**				
Superoxide dismutase (SOD)	f = a*exp(-,5*((x-x0)/b)^2)	Octanoic acid	96.38 (82.77 -109.99)	2.71 (2.14-3.28)	3.15 (2.75-3.56)	14	29.72	<0.0001	0.83
		Octanoir acid + *C. lunata*	91.27 (79.35-103.19)	1.97 (1.69-2.26)	3.60 (3.37-3.82)	14	63.22	<0.0001	0.91
	F=y0-ax	*M. citrifolia*	2.45 (3.04-1.87)	-	28.93 (26.45-31.41)	14	81.95	<0.0001	0.86
		M. citrifolia + *C. lunata*	4.96 (6.43-3.50)	-	46.85 (40.67-53.04)	14	53.88	<0.0001	0.81
Catalase (CAT)	f = a*exp(-b*x)	Octanoic acid	59.88 (52.21-67.54)	0.41 (0.32-0.50)	-	14	262.17	<0.0001	0.95
		Octanoir acid + C. lunata	2.31 (2.99-1.62)	-	29.32 (26.42-32.22)	14	52.96	<0.0001	0.96
	F=y0-ax	*M. citrifolia*	1.52 (2.12-0.93)	-	22.33 (19.81-24.85)	14	30.58	<0.0001	0.70
		M. citrifolia + *C. lunata*	1.60 (2.24-0.97)	-	18.76 (16.06-21.46)	14	29.61	0.0001	0.70
Ascorbate peroxidase (APX)	F=y0-ax	Octanoic acid	8.31 (11.68 -4.94)	-	197.24 (182.99-211.49)	14	28.38	0.0001	0.69
		Octanoir acid + *C. lunata*	6.44 (8.44-4.44)	-	161.28 (152.83-169.74)	14	48.40	<0.0001	0.79
		M. citrifolia	-	-	-	-	-	-	-
	f = y0+ax+bx^2	*M. citrifolia* + *C. lunata*	42.95 (57.99-27.91)	6.26 (4.45-8.08)	254.30 (232.34-276.27)	14	41.47	<0.0001	0.87
Phenol peroxidase (POX)	F=y0-ax	Octanoic acid	0.05 (0.07-0.037)	-	0.99 (0.91-1.08)	14	37.25	<0.0001	0.74
	f = a*exp(-,5*((x-x0)/b)^2)	Octanoir acid + *C. lunata*	1.15 (0.94-1.37)	3.26 (2.30-4.22)	4.28 (3.66-4.90)	14	8.56	0.0049	0.58
	F=y0+ax	*M. citrifolia*	0.02 (0.01-0.03)	-	0.13 (0.10-0.16)	14	49.72	<0.0001	0.79
	f = a*exp(-,5*((x-x0)/b)^2)	M. citrifolia + *C. lunata*	1.39 (1.28-1.49)	1.56 (1.44-1.67)	3.01 (2.89-3.12)	14	221.37	<0.0001	0.97
Quitinase (QUIT)	f = a*exp(-,5*((x-x0)/b)^2)	Octanoic acid	1.86 (1.70-2.02)	4.17 (3.24-5.09)	3.22 (2.71-3.72)	14	19.42	0.0002	0.76
		Octanoir acid + *C. lunata*	2.87 (2.67-3.07)	2.66 (2.42-2.91)	3.63 (3.45-3.81)	14	92.32	<0.0001	0.93
		M. citrifolia	-0.16 (-0.22-0.10)	-	1.81 (1.57-2.05)	14	39.29	<0.0001	0.75
	f=y0-ax	*M. citrifolia* + *C. lunata*	-	-	-	-	-	-	-

Table S2. Target model of fungi used to analyze the molecular docking with the major compounds of *Morinda citrifolia* essential oil.

Organism	Target (Unipot database)	Template	Identidade (%)	Ramachandran Favoured (%)	QMEAN
Curvularia lunata similarity *Cochliobolus sativus*	Tyrosine- tRNA ligase (M2TAQ3)	4Q93	52.89%	95.04%	-0.73

Table S3. Molecular Docking results for complexes between major compounds of *Morinda citrifolia* essential oil and target of fungi.

**Ligand**	**Organism**<br>***Curvularia lunata*** **related**<br>**Kcal/mol***
Octanoic acid	-5.0
Hexanoic acid	-4.2
Hexanoic acid, 4-pentenyl ester (pent-4-enyl hexanoate)	-4.6
Octanoic acid, methyl ester (methyl octanoate)	-4.4
Hexanoic acid, methyl ester (methyl hexanoate)	-4.1
Isobutyl 3-methylbut-3-enyl carbonate<br>(3-methylbut-3-enyl 2-methylprpyl carbonate)	-4.6

*Affinity energy

Figure S2: Molecular dynamic simulation from *Curvularia lunata* related with ligand octanoic acid.

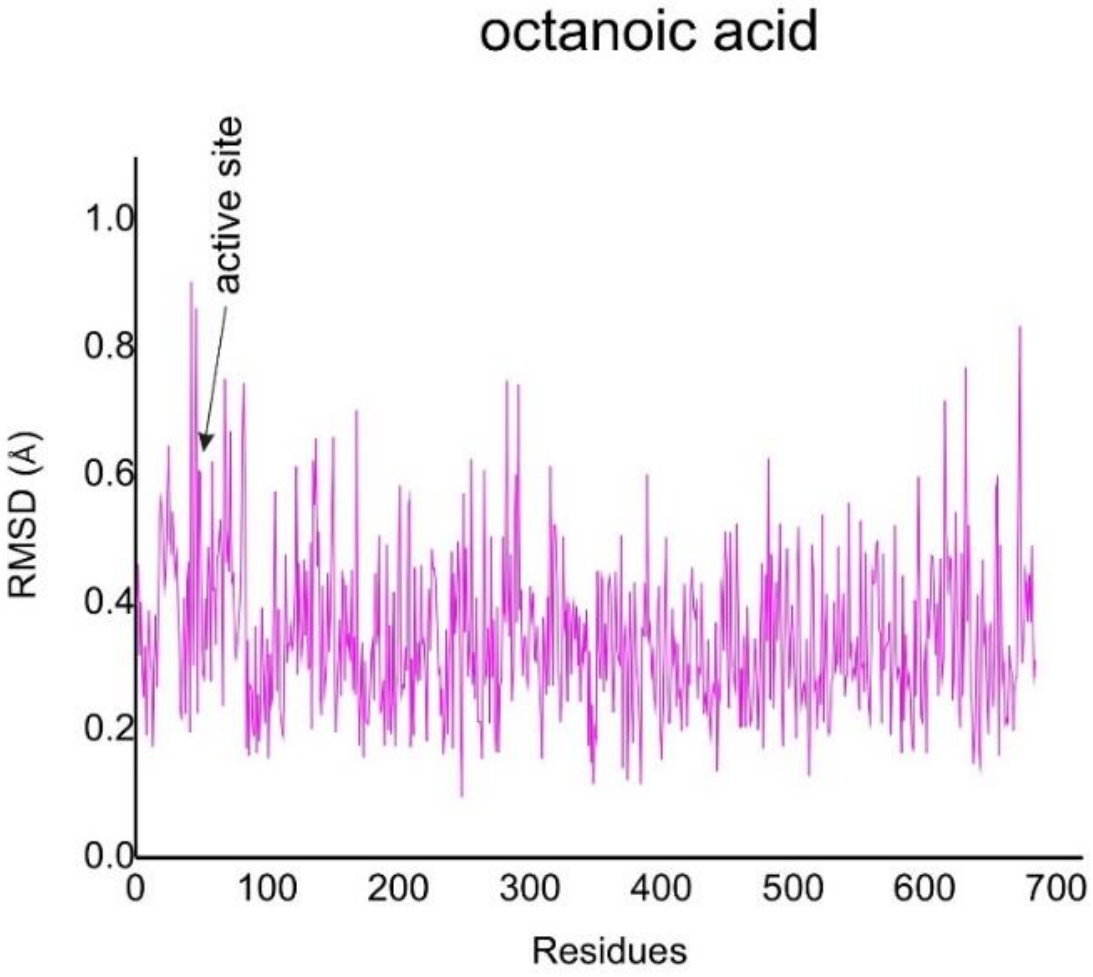

Table S4. Repeated measures analysis of variance (ANOVA) between subjects for the insecticide repellence to adults and the Stadio vegetative of maize plant.

Variation source		*df*	F	*P*	
between subjects					
Insecticide (I)		2	8.63	**0.0007**	
Vegetative stage (Vs)		1	632.32	**<0.0001**	
I vs Vs		2	1.02	**0.0006**	
Error		42	-	-	
Within subject effects	df_{den}	df_{num}	Wilks' lambda	F	*P*
Time (T)	39.00	4	0,346	18,38	**<0.0001**
T vs I	78	8	0.872	0.69	0.7002
T vs Vs	39	4	0.393	15.04	**<0.0001**
T vs I vs S	78	8	0.845	0.85	0.5580
